# The fatty acid elongase Elovl6 is crucial for hematopoietic stem cell engraftment and leukemia propagation

**DOI:** 10.1038/s41375-023-01842-y

**Published:** 2023-03-08

**Authors:** Yusuke Kiyoki, Takayasu Kato, Sakura Kito, Takashi Matsuzaka, Shin Morioka, Junko Sasaki, Kenichi Makishima, Tatsuhiro Sakamoto, Hidekazu Nishikii, Naoshi Obara, Mamiko Sakata-Yanagimoto, Takehiko Sasaki, Hitoshi Shimano, Shigeru Chiba

**Affiliations:** 1 Graduate School of Comprehensive Human Sciences, University of Tsukuba, 1-1-1 Tennodai, Tsukuba, Ibaraki 305-8575 Japan; 2 Department of Hematology, Faculty of Medicine, University of Tsukuba, 1-1-1 Tennodai, Tsukuba, Ibaraki 305-8575 Japan; 3 Department of Laboratory Medicine, Faculty of Medicine, University of Tsukuba, 1-1-1 Tennodai, Tsukuba, Ibaraki 305-8575 Japan; 4 Transborder Medical Research Center, University of Tsukuba, 1-1-1 Tennodai, Tsukuba, Ibaraki 305-8575 Japan; 5 Department of Endocrinology and Metabolism, Faculty of Medicine, University of Tsukuba, 1-1-1 Tennodai, Tsukuba, Ibaraki 305-8575 Japan; 6 Department of Biochemical Pathophysiology, Medical Research Institute, Tokyo Medical and Dental University, 1-5-45 Yushima, Bunkyo-ku, Tokyo 113-8510 Japan; 7 Department of Lipid Biology, Graduate School of Medical and Dental Sciences, Tokyo Medical and Dental University, 1-5-45 Yushima, Bunkyo-ku, Tokyo 113-8510 Japan; 8 Department of Cellular and Molecular Medicine, Graduate School of Medical and Dental Sciences, Tokyo Medical and Dental University, 1-5-45 Yushima, Bunkyo-ku, Tokyo 113-8510 Japan; 9 Division of Advanced Hemato-Oncology, Transborder Medical Research Center, University of Tsukuba, 1-1-1 Tennodai, Tsukuba, Ibaraki 305-8575 Japan

**Keywords:** Acute myeloid leukaemia, Haematopoietic stem cells, Cancer metabolism

## To the Editor

Lipid metabolism plays an essential role in regulating stem [1] and cancer [2] cell function. The biological significance of lipid species diversity, termed ‘lipid code’, has drawn increasing attention. Variations in the length and degree of desaturation of fatty acid (FA) chains contribute to this diversity. Among the rate-limiting enzymes catalyzing long-chain FA elongation [elongation of very long-chain fatty acid proteins (ELOVL1–7)], ELOVL6 converts C16 saturated and monounsaturated FAs to C18 species, the most important step for the de novo synthesis of endogenous long-chain FAs [3]. *Elovl6* gene disruption (*E6KO*) in mice decreases the proportion of stearate (C18:0) and oleate (C18:1n-9) and increases that of palmitate (C16:0) and palmitoleate (C16:1n-7) in the liver [3, 4]. *E6KO* ameliorates metabolic and inflammatory diseases in mice such as insulin resistance [3] and non-alcoholic steatohepatitis [5]. Moreover, high ELOVL6 expression correlates with poor prognosis of patients with breast [6] and liver [7] cancer.

Here, we report that ELOVL6 is essential for hematopoietic stem cell (HSC) engraftment after bone marrow (BM) transplantation and that it blocks acute myeloid leukemia (AML) development in a mouse model. These outcomes are at least in part attributed to the defective chemotaxis of HSCs or of transformed hematopoietic progenitor cells (HPCs) owing to diminished CXCL12 signaling through the PI3K-RAC pathway.

**Fig. 1:**
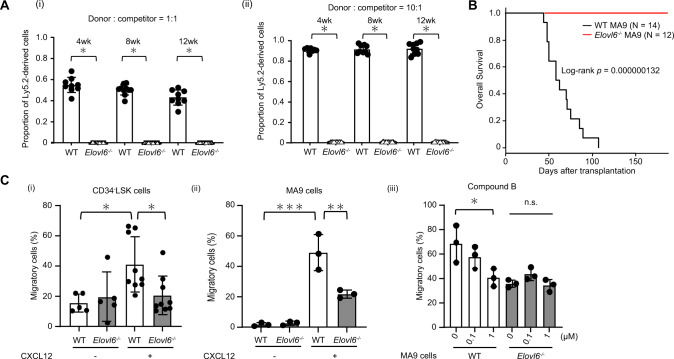
*Elovl6* is essential for hematopoietic stem cell (HSC) repopulation and acute myeloid leukemia (AML) propagation by *MLL::AF9*-immortalized (MA9) cells in mice. **A** Results of competitive repopulation assay. Shown are PB chimerism of Ly5.2 cells after transplantation with 1 × 10^6^ Ly5.2 WT (*n* = 9) or *E6KO* (*n* = 15) test cells and 1 × 10^6^ Ly5.1/5.2 competitor BM cells (i) or transplantation with 2 × 10^6^ Ly5.2 WT (*n* = 9) or *E6KO* (*n* = 15) test cells and 2 × 10^5^ Ly5.1/5.2 competitor BM cells (ii). **B** Survival of mice transplanted with WT or *E6KO* MA9 cells. Representative data of three independent experiments are shown. **C** Transwell chemotaxis assay. Percent migration of WT or *E6KO* CD34^-^LSK cells (control; *n* = 5 each. CXCL12; *n* = 9 each.) (i) and WT or *E6KO* MA9 cells (*n* = 3 each) (ii) toward CXCL12-containing or control chambers. Effect of ELOVL6 inhibitor on migration of WT or *E6KO* MA9 cells toward a CXCL12-containing chamber; *n* = 3 each (iii). Data are shown as the mean ± SD in all bar charts. *P < 0.05, **P < 0.01, ***P < 0.001.

We first found that *Elovl6* transcripts levels were higher in HSC and HPC fractions than in most peripheral blood cell fractions (Fig. S1A, B). Then, we analyzed FAs in BM cell lysates and observed significantly increased palmitate levels and decreased stearate to palmitate ratio in *E6KO* cells, relative to that in wild-type (WT) cells, as seen in the liver of *E6KO* mice [3] (Fig. S1C). To assess HSC function following *E6KO*, we performed competitive repopulation assays at a 1:1 ratio between WT or *E6KO* donor cells (Ly5.2) and WT competitors (Ly5.1/Ly5.2). Notably, in the recipient mice, percentage of donor-derived BM cells was around 50% and <0.2% with WT and *E6KO* Ly5.2 cells, respectively, at 12 weeks after transplantation. Even at a ratio of 10:1, the percentage of donor-derived *E6KO* BM cells was similarly low while that of WT Ly5.2 cells was >90% (Fig. 1A(i), (ii), and S1D, E). Next, we explored the effect of *E6KO* on leukemia transformation and propagation using the HPCs from *E6KO* mice. We retrovirally transduced MLL::AF9 into lineage-negative and c-Kit^+^ cells from WT or *E6KO* mouse BM. The resulting cells (MA9 cells) of both genotypes proliferated exponentially in a comparable manner [8, 9] (Fig. S2A). Subsequently, we transplanted lethally irradiated syngeneic mice with either WT or *E6KO* MA9 cells (Fig. S2B). All mice receiving WT cells developed AML as previously reported [9]; however, AML propagation was blocked in *E6KO* MA9 cell-transplanted recipients (Fig. 1B).

To determine the mechanisms underlying *E6KO* HSC and MA9 cell engraftment failure, we performed whole transcriptome analysis with CD34^-^LSK and MA9 cells of WT or *E6KO* backgrounds. This analysis demonstrated enrichment of chemotaxis and CXCR4 pathway gene sets in *E6KO* MA9 cells, relative to those in WT cells (Fig. S3A). To substantiate these results, we tested in vitro chemotaxis of *E6KO* CD34^-^LSK and MA9 cells toward CXCL12 in a chemotaxis assay (Fig. 1C). A greater fraction of WT than that of *E6KO* CD34^-^LSK cells migrated to the CXCL12-containing chamber, with specific chemotaxis of ~20% and ~0%, respectively (Fig. 1C(i)). In WT and *E6KO* MA9 cells, the fraction of cells showing specific chemotaxis toward CXCL12 was also greater in WT cells than in *E6KO* MA9 cells (Fig. 1C(ii)), while CXCR4 expression levels on WT and *E6KO* MA9 cells were comparable (Fig. S3B). To determine whether inhibition of ELOVL6 affects the migratory ability of MA9 cells, we performed a chemotaxis assay using Compound B, an ELOVL6 inhibitor. Chemotaxis was decreased in a Compound B concentration-dependent manner in WT MA9 cells. By contrast, Compound B did not affect the migration of *E6KO* MA9 cells (Fig. 1C(iii)). To verify these findings in HSCs in vivo, we connected Ly5.1 WT mice using parabiotic surgery one by one to WT or *E6KO* Ly5.2 mice (Fig. S3C). Four weeks later, 0.8% of LSK and 1.6% of CD34^-^LSK cells, on average, in the BM of Ly5.1 mice, were replaced with Ly5.2 cells from WT mice, while almost no LSK or CD34^-^LSK cells were replaced with Ly5.2 cells from *E6KO* mice (Fig. S3C). We then analyzed homing capacity of MA9 cells in the recipient BM at 48 h after transplantation, finding a proportion of WT MA9 cells detectable but *E6KO* MA9 cells undetectable (Fig. S3D).

**Fig. 2:**
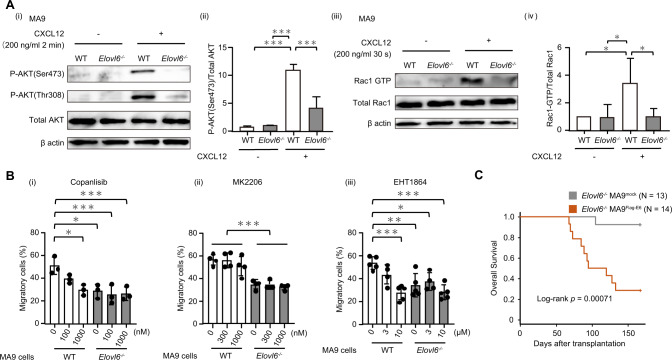
ELOVL6 regulates chemotaxis via PI3K-RAC signaling following CXCL12 stimulation and is involved in mouse AML. **A** Phosphorylation of AKT in wild-type (WT) or *E6KO* MA9 cells before and after CXCL12 stimulation. Representative western blot (i) and results of three experiments (ii). Pull-down assay for GTP-bound Rac1 in WT or *E6KO* MA9 cells before and after CXCL12 stimulation. Representative western blot (iii) and results of four experiments (iv). **B** Effect of Copanlisib (i), MK2206 (ii), and EHT1864 (iii) on migration of WT or *E6KO* MA9 cells toward a CXCL12-containing chamber; *n* = 3–5 each. **C** Survival of mice transplanted with *E6KO* MA9^Flag-E6^ and *E6KO* MA9^mock^ cells.

CXCR4 signaling depends in part on PI3K activating the downstream cascades. To determine whether *Elovl6* loss alters PI3K activation, we examined AKT phosphorylation, a representative readout of PI3K activity [10], in WT and *E6KO* MA9 cells before and after CXCL12 stimulation. CXCL12 stimulation increased AKT phosphorylation at Ser473 and Thr308 in MA9 cells of both genotypes, but these increases were significantly weaker in *E6KO* cells than in WT MA9 cells (Fig. 2A(i), (ii)). ERK phosphorylation, which is induced by CXCR4 activation, via a signaling pathway different from PI3K [11], was also induced by CXCL12 stimulation but was unaffected by *Elovl6* loss (Fig. S4A). Gene set enrichment analysis indicated that Rac1 pathway molecules were more enriched in *E6KO* cells than in WT MA9 cells (Fig. S3A(v)) and that Rac1 is reportedly activated by PI3K downstream [12]. We, thus, investigated whether Rac1 activation is blocked by *Elovl6* loss. Levels of GTP-bound Rac1 markedly increased in WT MA9 cells following CXCL12 stimulation, but the increase was not significant in *E6KO* MA9 cells (Fig. 2A(iii), (iv)).

We then examined the pharmacologic effects of inhibiting PI3K, AKT, or RAC pathways on chemotaxis. In a transwell assay, the presence of the pan-PI3K inhibitor copanlisib or pan-RAC inhibitor EHT1864 decreased the fraction of WT MA9 cells migrating to the CXCL12-containing chamber. However, *E6KO* MA9 cell migration, which was already compromised, was not significantly affected by copanlisib or EHT1864. By contrast, the pan-AKT inhibitor MK2206 did not alter WT or *E6KO* MA9 cell migration (Fig. 2B(i)–(iii)), suggesting minimal effects of AKT activation on MA9 cell motility.

Given impaired chemotaxis and PI3K-Rac1 signaling in *Elovl6*-deficient cells, we investigated whether these cells exhibited altered actin remodeling in response to CXCL12. CXCL12 stimulation increased the fraction of WT CD34^-^LSK cells showing lamellipodia from 14 to 52% (Fig. S4F(i), (ii)), whereas the fraction of *E6KO* CD34^-^LSK cells exhibiting lamellipodia remained unchanged (Fig. S4F(ii)). Thus, ELOVL6 is likely to regulate a pathway that is responsive to CXCL12, resulting in PI3K-Rac1-mediated cytoskeletal remodeling and chemotaxis.

To determine whether exogenously expressed *Elovl6* could rescue *E6KO* MA9 cell phenotypes, we generated *E6KO* MA9 cells expressing Flag-tagged ELOVL6 (*E6KO* MA9^Flag-E6^) and mock controls (*E6KO* MA9^mock^) (Fig. S5A). First, we evaluated AKT phosphorylation (Fig. S5B) and Rac1 activation (Fig. S5C) following CXCL12 stimulation. Levels of both were higher in *E6KO* MA9^Flag-E6^ cells than in *E6KO* MA9^mock^ cells and comparable to the levels seen in WT MA9^Flag-E6^ and WT MA9^mock^ cells. In chemotaxis analysis, the migration ratio was increased in *E6KO* MA9^Flag-E6^ cells compared to that seen with *E6KO* MA9^mock^ cells (Fig. S5D). Finally, to evaluate rescue of AML propagation, we transplanted lethally irradiated syngeneic mice with either *E6KO* MA9^mock^ or *E6KO* MA9^Flag-E6^ cells and observed that among the recipient mice transplanted with *E6KO* MA9^mock^ cells, very few recipients developed AML, as with *Elovl6*^*−/−*^ MA9 cells (Fig. 2C). By contrast, 10 out of 14 recipients died due to AML following *E6KO* MA9^Flag-E6^ cell transplantation (Fig. 2C), suggesting the re-acquisition of leukemogenic potential following *Elovl6* re-expression.

To gain an insight into whether ELOVL6 expression levels show any clinical significance in AML patients, we used GEPIA2 (ref. [13]) for The Cancer Genome Atlas cohort. This analysis revealed the association between high *ELOVL6* expression levels and worse overall survival (Fig. S6A). Similar results were obtained from the Beat AML data (Fig. S6B).

In summary, this study reports novel outcomes in normal HSCs and transformed hematopoietic cells following lipid metabolic changes induced by *Elovl6* deficiency. Our finding that *Elovl6* loss hampers AML propagation will facilitate the development of novel cancer treatments; ELOVL6 activity or pathways regulated by ELOVL6 are potential targets of anti-AML therapy.

## Supplementary information

Supplementary Information for 'The fatty acid elongase Elovl6 is crucial for hematopoietic stem cell engraftment and leukemia propagation'

Supplementary Table 2

Supplementary Table 3

## Data Availability

Further information and requests for resources and reagents including cell lines and mouse models should be directed to and will be fulfilled by TK and SC.
