# Supplementary material for: The fatty acid elongase Elovl6 is crucial for hematopoietic stem cell engraftment and leukemia propagation
Source: Leukemia. 2023 Mar 8;37(4):910–3. doi: 10.1038/s41375-023-01842-y (PMC10079543; doi:10.1038/s41375-023-01842-y)
Supplement: Supplementary file 1 — Supplementary Information for 'The fatty acid elongase Elovl6 is crucial for hematopoietic stem cell engraftment and leukemia propagation' [file 41375_2023_1842_MOESM1_ESM.pdf]

Supplementary Information for

The fatty acid elongase Elovl6 is crucial for hematopoietic stem cell engraftment and leukemia propagation

Yusuke Kiyoki[1*], Takayasu Kato[2, 3*], Sakura Kito[1], Takashi Matsuzaka[4, 6], Shin Morioka[7, 8], Junko Sasaki[7, 8, 9], Kenichi Makishima[1], Tatsuhiro Sakamoto[2], Hidekazu Nishikii[2], Naoshi Obara[2], Mamiko Sakata-Yanagimoto[2, 5], Takehiko Sasaki[7, 8], Hitoshi Shimano[6], and Shigeru Chiba[2]

1) Graduate School of Comprehensive Human Sciences, University of Tsukuba, 1-1-1 Tennodai, Tsukuba, Ibaraki 305-8575, Japan
2) Department of Hematology, Faculty of Medicine, University of Tsukuba, 1-1-1 Tennodai, Tsukuba, Ibaraki 305-8575, Japan
3) Department of Laboratory Medicine, Faculty of Medicine, University of Tsukuba, 1-1-1 Tennodai, Tsukuba, Ibaraki 305-8575, Japan
4) Transborder Medical Research Center, University of Tsukuba, 1-1-1 Tennodai, Tsukuba, Ibaraki 305-8575, Japan
5) Division of Advanced Hemato-Oncology, Transborder Medical Research Center, University of Tsukuba, 1-1-1 Tennodai, Tsukuba, Ibaraki 305-8575, Japan
6) Department of Endocrinology and Metabolism, Faculty of Medicine, University of Tsukuba, 1-1-1 Tennodai, Tsukuba, Ibaraki 305-8575, Japan
7) Department of Biochemical Pathophysiology, Medical Research Institute, Tokyo Medical and Dental University, 1-5-45 Yushima, Bunkyo-ku, Tokyo 113-8510, Japan
8) Department of Lipid Biology, Graduate School of Medical and Dental Sciences, 1-5-45 Yushima, Tokyo Medical and Dental University, Bunkyo-ku, Tokyo 113-8510, Japan
9) Department of Cellular and Molecular Medicine, Graduate School of Medical and Dental Sciences, Tokyo Medical and Dental University, 1-5-45 Yushima, Bunkyo-ku, Tokyo 113-8510, Japan
*These authors contributed equally to this work.

Corresponding authors:
Takayasu Kato and Shigeru Chiba
Department of Hematology, Faculty of Medicine, University of Tsukuba, 1-1-1 Tennodai, Tsukuba, Ibaraki 305-8575, Japan

Phone: +81-29-853-3103, Fax: +81-29-853-8079
Email: tkato@md.tsukuba.ac.jp and schiba-t@md.tsukuba.ac.jp

This PDF file includes:

Methods
Supplementary Results
Supplementary Discussion
Supplementary Figures 1-8
Supplementary Tables 1-3

Methods

Mice

Elovl6$^{-/-}$ mice were generated as previously described[1]. Young adult (8–16 weeks) C57BL/6 (B6)-Ly5.2 and B6-Ly5.1 mice were purchased from Sankyo Labo Service Corporation (Tokyo, Japan). Mice were housed at the Animal Center for Biomedical Research, University of Tsukuba, following institutional guidelines. Our animal study protocols were approved by the relevant institutional committee (approval number: 22-374).

Flow cytometry

Before analysis, mouse BM cells were flushed from the femur and tibia of 8–16-week-old mice. For purification of c-Kit positive and lineage marker-negative cells, cells were stained with allophycocyanin (APC)-conjugated anti-c-Kit antibody, followed by incubation with APC MicroBeads. c-Kit positive cells were manually separated using MS columns and incubated for 30 min with a lineage antibody mixture consisting of biotinylated anti-Gr-1, anti-Mac-1, anti-B220, anti-CD4, anti-CD8, anti-CD127, and anti-TER119 antibodies at 4 °C. After washing with phosphate-buffered saline (PBS), cells were further stained with APC-conjugated anti-c-Kit antibody at 4 °C for an additional 30 min. Multicolor analysis and sorting were performed with a FACSAria II/III (BD Biosciences, San Jose, CA, USA).

Quantitative PCR with reverse transcription (RT-qPCR)

To confirm *Elovl6* expression pattern in the hematopoietic compartment, we accessed *Elovl6* expression in whole PB and BM cells and in flow cytometry-separated lineage-negative (Lin$^-$) cells; megakaryocyte-erythroid progenitors; granulocyte-monocyte progenitors; common myeloid progenitors; Lin$^-$, c-Kit$^+$, and Sca1$^+$ (LSK) cells; CD34$^-$LSK cells from BM of WT mice. RNA was isolated from blood cells using an RNeasy kit (QIAGEN, Valencia, CA, USA). cDNA was synthesized with SuperScript II (Invitrogen, Carlsbad, CA, USA) according to the

manufacturer's instructions. Reaction products were diluted in distilled water, of which 5-μL aliquots were used for real-time PCR.

Expression levels of analyzed transcripts were detected with the SYBR green reagent (Roche Diagnostics, Basel, Switzerland) using an ABI 7500 apparatus (Thermo Fisher Scientific, Waltham, MA, USA)). Gene primers are listed in Supplementary Table 1.

Competitive repopulation assays

Using WT or Elovl6$^{-/-}$ BM cells, nucleated BM cells (Ly5.2) from each genotype were mixed in a 1:1 or 10:1 ratio with 1×10^6 competitor BM cells (Ly5.1/Ly5.2) and injected into lethally irradiated (9.5 Gy) recipients (Ly5.1). Ly5.2 chimerism in recipients was measured in PB samples at the indicated time points post-transplantation.

fatty acids (FA) composition analysis

Lipids from BM cells and MA9 cells ($1–5 \times 10^6$ cells/sample) were extracted as previously described[2]. Briefly, cells were extracted using a chloroform/methanol (1:2, v/v) solution. After homogenization for 2 min, samples were centrifuged at 1,500 × *g* for 5 min at 22 °C. The aqueous solution was discarded, and the lipid-containing chloroform phase was transferred to a test tube. The chloroform was evaporated using nitrogen gas and the extracted lipids were sent to an external laboratory (SRL Inc., Tokyo, Japan) for FA quantification using gas chromatography.

Parabiosis

Pairs of 6–8-week-old and weight-matched female Ly5.1-WT and Ly5.2-Elovl6$^{-/-}$ mice were housed together for 1–2 weeks, then underwent parabiotic surgery using the methods adapted from Bunster and Meyer[3]. Mice were anesthetized with 0.3 mg/kg of medetomidine (Kyoritsu Pharma, Tokyo, Japan), 4.0 mg/kg midazolam (Astellas Pharma, Northbrook, IL, USA), and 5.0 mg/kg of butorphanol (Meiji Seika Pharma, Tokyo, Japan) as previously described[4]. Operative sides were shaved and cleaned for sterility. Lateral skin was opened from knee to shoulder and

freed of attached tissue. Opposing muscle and perineum was sutured with 4-0 chromic gut (vital sutures), and corresponding skin was joined with 3-0 Vicryl (Johnson & Johnson, New Brunswick, NJ, USA). One hundred microliters of blood sample was collected from the orbital sinus (after sedation with a 3% isoflurane inhalant anesthetic) to document joint circulation via flow cytometry of WBCs. After 4 weeks, mice were anesthetized with tribromoethanol, sacrificed by cervical dislocation, and separated through transection at the anastomosis site. Before separation, 0.2 mL of blood was obtained by eye puncture. After separation, 4–6 × 10^7 marrow cells were obtained by dissecting the femurs.

Colony-forming assay

Single $CD34^-$LSK cells was sorted into 96-well plates using a FACS-based automated cell deposition unit and cultured in StemSpan SFEM supplemented with 20 ng/ml mouse SCF, 20 ng/mL human TPO, 20 ng/ml mouse IL-3, and 10 IU/mL human EPO. After 14 d of culturing, colony numbers were determined.

Vectors

MLL::AF9 cDNA in a retrovirus vector, pGCDNsam/IRES-GFP, has been previously described[5]. The C-terminal FLAG-tagged *Elovl6* was constructed using a PrimeSTAR Mutagenesis Basal kit (Takara Bio Inc. Shiga, Japan) and subcloned into a pGCDNsam/IRES-humanized Kusabira orange (hKO) vector.

Generation of the MLL::AF9 AML model

MLL::AF9 retroviruses were generated in 293GP packaging cells transfected with the MSCV-MLL::AF9- IRES-GFP plasmid using calcium phosphate co-precipitation as previously described[5]. Viral supernatants were harvested after 48–72 h, filtered through a 0.45-µm membrane, and concentrated via centrifugation at 6,000 × *g* for 16 h at 4 °C. Retroviruses were used to infect BM lineage-negative and c-kit positive cells derived from WT and $Elovl6^{-/-}$ mice, and loaded onto 50 µg/mL retronectin-coated 96-well plates and cultured for one week with

IMDM supplemented with 10% fetal calf serum (FCS), 50 ng/mL mouse recombinant stem cell factor (SCF), 10 ng/mL interleukin (IL)-3, 10 ng/mL IL-6, 10 ng/mL murine thrombopoietin (mTPO), and 1% penicillin/streptomycin. Sorted GFP-positive cells were cultured with RPMI-1640 supplemented with 10% FCS, 10 ng/mL IL-3, and penicillin/streptomycin. MA9 cells (1×10^6) were transplanted by injection into the tail vein of lethally irradiated (950 cGy) syngeneic C57BL/6 mice, together with 2.5×10^5 whole BM cells collected from Ly5.2 mice as radioprotection. To generate *Elovl6* cDNA re-expressed MA9 cells, GFP-positive WT or *E6KO* cells were infected with a retroviral vector carrying FLAG-tagged *Elovl6* cDNA and expressing humanized Kusabira orange (hKO) or mock-hKO. GFP^+/hKO^+ double-positive MA9 cells were sorted and cultured. Lysates were immunoblotted with anti-FLAG antibody to confirm *Elovl6* cDNA re-expression.

Proliferation assay

MA9 cells were seeded at a density of 1×10^4 cells/well on 96-well plates and cultured in RPMI-1640 containing 10% FCS and 10 ng/mL IL-3. Proliferation was evaluated by counting cells daily using a hemocytometer. All assays were evaluated in at least three independent experiments.

Preparation of FA solution

Oleic acids were dissolved to 100 mM in ethanol heated at 70 °C to make stock solutions for later dilution in RPMI medium supplemented with 0.5% FA-free BSA and 10% FCS to a final concentration of 0–1,000 µM. MA9 cells were treated for 1 week before chemotaxis assay.

Western blotting

MA9 cells (2×10^5) were washed twice with PBS and then treated with 200 ng/mL CXCL12 for 2 min or an inhibitor (copanlisib or MK2206) for 4 h at 37 °C and 5% CO_2. The cells were then lysed using Tris-base saline Tween 20 (TBST: 40 mM Tris-HCl, pH 7.4; 100 mM NaCl, 2 mM EDTA; 1% Tween 20) supplemented with a protease inhibitor cocktail (Roche Diagnostics) and

boiled in Laemmli's buffer. These lysates were then subjected to 10–12% SDS-PAGE. Polyvinylidene fluoride membranes were blocked in 5% BSA in TBST at 22–25 °C for 1 h and incubated overnight at 4 °C in TBST supplemented with primary antibodies (dilutions according to the manufacturer's recommendations), as listed in Supplemental Table 2. Then, the membranes were washed with TBST and treated with a secondary antibody (1:10,000 in TBST) at 22 °C for 1 h. Protein signal band intensity was measured using ImageQuant LAS-4010.

Bioinformatics analysis of gene expression

Clinical data and *ELOVL6* mRNA expression level of 446 primary AML samples were downloaded from Vizome (http://www.vizome.org). For survival analysis, 446 patients were divided into two groups (low and high expression) using expression cut-off values calculated using the Youden's index[6].

mRNA sequencing

The RNA-Seq method used herein has been previously described[7]. Briefly, total RNA was extracted from $CD34^{-}$LSK (WT: n = 2, *Elov6*$^{-/-}$: n = 4) and immortalized MA9 (WT: n = 2, Elovl6$^{-/-}$: n = 2) cells using the TRIzol reagent (Thermo Fisher Scientific, Waltham, MA, USA), according to the manufacturer's instructions. The RNA-Seq library was prepared using a NEBNext Ultra Directional RNA Library Prep Kit (New England Biolabs [NEB], Ipswich, MA) after depletion of rRNA (NEBNext rRNA Depletion Kit; NEB). Paired-end sequencing (2 × 36 bases) was performed using the NextSeq500 platform (Illumina, SanDiego, CA, USA). FASTQ files were imported, and sequence reads were mapped using CLC Genomics Workbench (Version 10.1.1; QIAGEN, Redwood City, CA, USA). Differential gene expression was analyzed empirically using the Empirical Analysis of DGE tool (edgeR test) in the CLC Genomics Workbench and CLC Main Workbench. GSEA scores were generated for gene sets in the CURATED (C2) and GENE ONTOLOGY (C5) datasets using classic scoring, 1,000 gene-set permutations, and signal-to-noise metrics. The normalized enrichment scores, false

discovery rate, and FWER *P*-values were all calculated using the GSEA software (https://www.gsea-msigdb.org/gsea/index.jsp)[8].

The public web tool at Metascape (http://metascape.org/)[9] was used to identify significant pathways from RNA-Seq data.

Chemotaxis assay

Chemotaxis assays were performed using Transwell polycarbonate membranes (5.0-µM pore size) in 96-well plates. LSK cells (2×10^4) suspended in 0.2 mL Stem Span (STEMCELL Technologies, Vancouver, BC, Canada) and MA9 cells (2×10^5) suspended in 0.2 mL RPMI-1640 medium were placed in the insert. As a chemoattractant, serum-free medium containing 100 ng/mL CXCL12 was added to the lower chamber. After 4 h, cell migration was determined by counting cells in the bottom chamber using a hemocytometer or flow cytometry. When using an ELOVL6 inhibitor (Compound B)[10], MA9 cells were treated with vehicle (0.1% DMSO) or Compound B dissolved in 0.1% DMSO at 100–1,000 nM for 1 week at 37 °C and 5% CO_2. Compound B was added in the medium daily after washing the MA9 cells twice with PBS. Subsequently, MA9 cells were treated with vehicle (0.1% DMSO) or an inhibitor, including MK2006 (100–1,000 nM), copanlisib (300–1,000 nM), EHT1864 (3–10 µM), or Y-27362 (3–10 µM), for 4 h at 37 °C, 5% CO_2. All assays were evaluated in at least three independent experiments.

Immunofluorescence staining

Freshly isolated $CD34^-$LSK or MA9 cells were suspended in a droplet of serum-free PBS supplemented with or without 100 ng/mL CXCL12 (Peprotech, Rocky Hill, NJ, USA). Cells were plated on a poly-L-lysine-coated (Matsunami Glass Ind., Ltd. Osaka, Japan) glass slide and incubated at 37 °C for 30 min. After fixation with 2% paraformaldehyde, $CD34^-$LSK cells were incubated with Alexa Fluor 488-conjugated phalloidin (Abcam, Cambridge, Cambridgeshire, UK) and MA9 cells were incubated with Alexa Fluor 488-conjugated

phalloidin (Abcam) or Phalloidin-iFluor 555 (Abcam) at 22 °C for 30 min to label F-actin. After washing, stained slides were mounted using mounting medium with DAPI (VECTASHIELD, Vector Laboratories, Burlingame, CA, USA). Fluorescent images were acquired using a Leica TCS SP8 confocal microscope (Leica Microscopy System, Wetzlar, Germany) and a fluorescence microscope BZ-X700 (Keyence, Osaka, Japan).

Phosphoinositide analysis

After 6 weeks of culture in RPMI medium supplemented with 10% FCS and IL-3, 5×10^6 WT MA9 cells, *Elovl6*$^{-/-}$ MA9 cells, or *Elovl6*$^{-/-}$ MA9$^{Flag\text{-}E6}$ cells were washed with PBS twice, and cell pellets were immediately frozen in liquid nitrogen. Following the addition of 1.2 mL methanol, the pellet was homogenized by pipetting, transferred to a glass tube, and mixed with a solution of methanol (300 μL), ultrapure water (750 μL), 2 M HCl (750 μL), and 1 M NaCl (200 μL) containing 1 nmol C8:0/C8:0 PI(4,5)P_2 (as an absorption inhibitor), 20 pmol C17:0/C14:1 PI, and 10 pmol each of C17:0/C20:4 PI(4)P, C17:0/C20:4 PI(4,5)P_2, and C17:0/C20:4 PI (3–5)P_3 (internal standards). After vigorous vortexing, 3 mL $CHCl_3$ was added, and the mixture was vortexed for an additional 2 min. After centrifugation at 1,200 × *g* for 4 min at 22 °C, the lower organic phase containing the crude lipid extract was collected and transferred to a new glass tube. Pre-concentration and methylation reactions were performed before liquid chromatography–tandem mass spectrometry (LC-MS/MS), as previously described[11].

LC-MS/MS

LC-MS/MS was performed on an UltiMate 3000 LC system (Thermo Fisher Scientific) equipped with an HTC PAL autosampler (CTC Analytics, Zwingen, Switzerland) and a TSQ-Vantage (Thermo Fisher Scientific) using a multiple-reaction-monitoring experiment (MRM), as previously described[12]. MRM transitions are shown in Supplemental Table 3. Seventeen acyl variants of PI were calculated.

Statistical analysis

All *in vitro* experiments were performed at least in triplicate, and statistical analyses were performed using EZR software (Saitama Medical Center, Jichi Medical University, Saitama, Japan). The survival of transplanted mice was analyzed using the log-rank test, whereas other data were analyzed using the Student's *t*-test and one-way ANOVA with Bonferroni or Holm's test for multiple comparisons. *P*-values < 0.05 were considered to indicate significant differences.

Results

Blood cell counts, bone marrow HSPC frequencies, and colony-forming capacity of CD34$^-$ LSK cells in *E6KO* mice

To assess gross effects of *Elovl6* loss, we examined blood counts in WT and *E6KO* mice and observed comparable counts in both (Fig. S1F). Frequencies of mature myeloid cells in PB (Fig. S1G) or those of various HSC/HPC fractions in BM were also comparable between the genotypes (Fig. S1H). To determine the mechanisms underlying *E6KO* HSC engraftment failure, we examined the colony-forming capacity of CD34$^-$LSK cells from both genotypes and found that *E6KO* CD34$^-$LSK cells formed a relatively greater number of colonies than WT cells did (Fig. S1I), the inverse representation to the engraftment failure.

Peripheral blood analysis of recipient mice transplanted with MLL::AF9-transformed (MA9) cells

We infected lineage-negative and c-Kit$^+$ (LK) cells from WT mouse BM with leukemogenic MLL::AF9-IRES-GFP retrovirus, cultured them for one week in the presence of multiple cytokines, sorted GFP$^+$ cells, and grew them in the presence of interleukin (IL)-3. These cells (designated as WT MA9) proliferated exponentially, as previously described[5, 13]. Then, using the

same procedure, we established *E6KO* MA9 cells and found that WT and *E6KO* MA9 cells, grown for 4–8 weeks after sorting, proliferated at a comparable rate under standard culture conditions (Fig. S2A). All mice receiving WT MA9 cells developed AML within 16 weeks, showed >20% GFP^+ leukemia cells in peripheral blood (Fig. S2D(i)–(iii)), and eventually died (Fig. 1B). However, AML propagation in *E6KO* MA9 cell-transplanted recipients was blocked, and GFP^+ cells were absent in peripheral blood (Fig. S2D(ii) and (iii)). All these mice survived until 6 months after transplantation.

Transcriptome analysis with $CD34^-$LSK cells and MLL::AF9-transformed cells of WT and *E6KO* backgrounds

Whole transcriptome analysis of total RNA extracted from $CD34^-$LSK cells of WT or *E6KO* backgrounds was performed to assess differentially expressed genes using Gene Ontology (GO) via Metascape. Among the enrichment terms in GO (Fig. S1J) and specific biological processes (Fig. S1K), taxis and locomotion were the second most enriched, suggesting that *Elovl6* loss may alter HSC or HPC motility.

GO analyses with WT and *E6KO* MA9 cells identified terms similar to those that emerged for $CD34^-$LSK cells (Fig. S2E, F).

Fatty acid changes in MA-9 cells of WT and *E6KO* backgrounds

By evaluating FAs in WT and *E6KO* MA9 cells (Fig. S2C), we confirmed a decrease in the C18:0/C16:0 ratio in *E6KO* cells, relative to that in WT MA9 cells (Fig.S2C inset).

Pharmacologic effects of inhibiting PI3K, AKT, or ROCK pathway

Treatment of WT MA9 cells with the pan-PI3K inhibitor BAY 80-6946 (copanlisib) suppressed AKT (Ser473) phosphorylation in a dose-dependent manner (Fig. S4B), as did treatment with

the pan-AKT inhibitor MK2206 (Fig. S4C). In a Transwell assay, the presence of treatment of both WT and *E6KO* MA9 cells with Y-27632, a ROCK inhibitor, suppressed CXCL12-induced cell migration, suggesting that ROCK activation is critical for the general migration machinery but not specific to ELOVL6 function (Fig. S4D). In addition, *E6KO* MA9 cells cultured with oleic acids (300–1,000 μM) reverted their migratory ability toward CXCL12 (Fig. S4E).

Actin remodeling in MA9 cells

Lamellipodia formation was not apparent in either WT or *E6KO* MA9 cells following CXCL12 stimulation. However, WT MA9 cells formed clusters of actin filaments after CXCL12 stimulation (Fig. S4G(i)), and the number of WT MA9 cells showing such clusters in response to stimulation was significantly increased, which was not observed in *E6KO* MA9 cells (Fig. S4G(ii)). Thus, ELOVL6 may regulate a pathway that is responsive to CXCL12, resulting in PI3K-Rac1-mediated cytoskeletal remodeling and chemotaxis (Fig. S4H).

Reversion of FA composition and FA variation in PI and PIPs in *E6KO* MA9 cells by *Elovl6* re-expression

We generated *E6KO* MA9 cells expressing Flag-tagged ELOVL6 (*E6KO* $MA9^{Flag\text{-}E6}$) and mock controls (*E6KO* $MA9^{mock}$; Fig. S5A). To assess the rescue of ELOVL6 catalytic activity, we evaluated FAs in *E6KO* $MA9^{mock}$ and *E6KO* $MA9^{Flag\text{-}E6}$ cells and found a higher C18:0/C16:0 ratio in *E6KO* $MA9^{Flag\text{-}E6}$ than *E6KO* $MA9^{mock}$ cells. The C18:0/C16:0 ratio in *E6KO* $MA9^{Flag\text{-}E6}$ was comparable to those in WT $MA9^{Flag\text{-}E6}$ and WT $MA9^{mock}$ cells - two additional controls generated from WT MA9 cells (Fig. S7A).

We then measured FA variation separately for phosphatidylinositol (PI) and its phosphorylated species[11], given that PI(4,5)P_2 and PIP_3 are the respective substrate and product of PI3K. We collectively assessed PI, PIP_2 [total of PI(3,4)P_2, PI(3,5)P_2, and PI(4,5)P_2], and PIP_3 [PI (3–5)P_3] and observed that for PIP_2 and PIP_3 the amount of C36.1 (for two FAs) was decreased in *E6KO*

MA9, compared with that in WT MA9 cells and were rescued in *E6KO* MA9$^{Flag-E6}$ cells, compared with that in *E6KO* MA9 cells (Fig. S7B, C).

The actin cluster formation after CXCl12 stimulation was also assessed following *Elovl6* re-expression. The proportion of cells forming cluster of actin filaments was significantly increased in *E6KO* MA9$^{Flag-E6}$ cells compared to *E6KO* MA9^{mock} cells (Fig. S5E).

Discussion

In this study, *Elovl6* loss perturbs CXCL12-PI3K-Rac1 signaling and actin remodeling, thus impairing cell migration required for normal HSC activity and leukemogenesis (Fig. S8). ELOVL6 activity or a pathway regulated by ELOVL6 could be targeted as anti-AML therapy. Our findings suggest that the CXCL12-PI3K-Rac1 migration pathway is modulated by the changes in FA composition owing to *Elovl6* loss. The importance of the PI3K-RAC axis for intracellular actin regulation is well known[14] and supports our observation of impaired lamellipodia formation in HSCs by CXCL12 stimulation.

However, how *Elovl6*-mediated changes in FA composition modulate PI3K signaling remains unanswered. In this regard, our targeted lipidomics findings relevant to changes of FA composition in PIP_3, along with those in other PIs, provide interesting clues. We succeeded in detecting changes in PIP_3 FA composition and in rescuing those phenotypes by deletion and respective re-expression of *Elovl6*. Given the rescue of other phenotypes in *Elovl6*$^{-/-}$ cells reported here, we speculate that decreases in C36.1 PIP_3 proportions seen following *Elovl6* deficiency are crucial for proper signaling through the CXCL12-CXCR4-PIP_3 axis. These changes are likely reflected by increases in levels of C16 FAs and decreases in levels of C18 FAs following *Elovl6* loss. It remains unknown whether changes in the FA composition of PIP_3, which is a direct product of PI3K, alter PI3K signaling.

In summary, this study demonstrates novel and unexpected outcomes in normal HSCs and transformed hematopoietic cells following lipid changes induced by *Elovl6* deficiency.

References

1. Matsuzaka T, Shimano H, Yahagi N, Kato T, Atsumi A, Yamamoto T, et al. Crucial role of a long-chain fatty acid elongase, Elovl6, in obesity-induced insulin resistance. Nat Med. 2007;13:1193–1202.

2. Bligh EG, Dyer WJ. A rapid method of total lipid extraction and purification. Can J Biochem Physiol. 1959;37(8):911–917.

3. Kamran P, Sereti KI, Zhao P, Ali SR, Weissman IL, Ardehali R. Parabiosis in mice: a detailed protocol. J Vis Exp. 2013;6.

4. Kawai S, Takagi Y, Kaneko S, Kurosawa T. Effect of three types of mixed anesthetic agents alternate to ketamine in mice. Exp Anim. 2011;60:481–487.

5. Kato T, Sakata-Yanagimoto M, Nishikii H, Ueno M, Miyake Y, Yokoyama Y, et al. Hes1 suppresses acute myeloid leukemia development through FLT3 repression. Leukemia 2015;29:576–585.

6. Youden WJ. Index for rating diagnostic tests. Cancer 1950;3:32–35.

7. Hayashi T, Kudo T, Fujita R, Fujita SI, Tsubouchi H, Fuseya S, et al. Nuclear factor E2-related factor 2 (NRF2) deficiency accelerates fast fibre type transition in soleus muscle during space flight. Commun Biol. 2021;4:787.

8. Subramanian A, Tamayo P, Mootha VK, Mukherjee S, Ebert BL, Gillette MA, et al. Gene set enrichment analysis: a knowledge-based approach for interpreting genome-wide expression profiles. Proc Natl Acad Sci U S A 2005;102:15545–15550.

9. Zhou Y, Zhou B, Pache L, Chang M, Khodabakhshi AH, Tanaseichuk O, et al. Metascape provides a biologist-oriented resource for the analysis of systems-level datasets. Nat Commun. 2019;10:1523.

10. Shimamura K, Nagumo A, Miyamoto Y, Kitazawa H, Kanesaka M, Yoshimoto R*, et al.* Discovery and characterization of a novel potent, selective and orally active inhibitor for mammalian ELOVL6. Eur J Pharmacol. 2010;630:34–41.

11. Morioka S, Nakanishi H, Yamamoto T, Hasegawa J, Tokuda E, Hikita T, et al. A mass spectrometric method for in-depth profiling of phosphoinositide regioisomers and their disease-associated regulation. Nat Commun. 2022;13:83.

12. Koizumi A, Narita S, Nakanishi H, Ishikawa M, Eguchi S, Kimura H, et al. Increased fatty acyl saturation of phosphatidylinositol phosphates in prostate cancer progression. Sci Rep. 2019;9:13257.

13. Martin ME, Milne TA, Bloyer S, Galoian K, Shen W, Gibbs D*, et al.* Dimerization of MLL fusion proteins immortalizes hematopoietic cells. Cancer cell 2003;4:197–207.

14. Hu H, Juvekar A, Lyssiotis CA, Lien EC, Albeck JG, Oh D, et al. Phosphoinositide 3-Kinase Regulates Glycolysis through Mobilization of Aldolase from the Actin Cytoskeleton. Cell 2016;164:433–446.

Supplementary Figures

Figure S1.

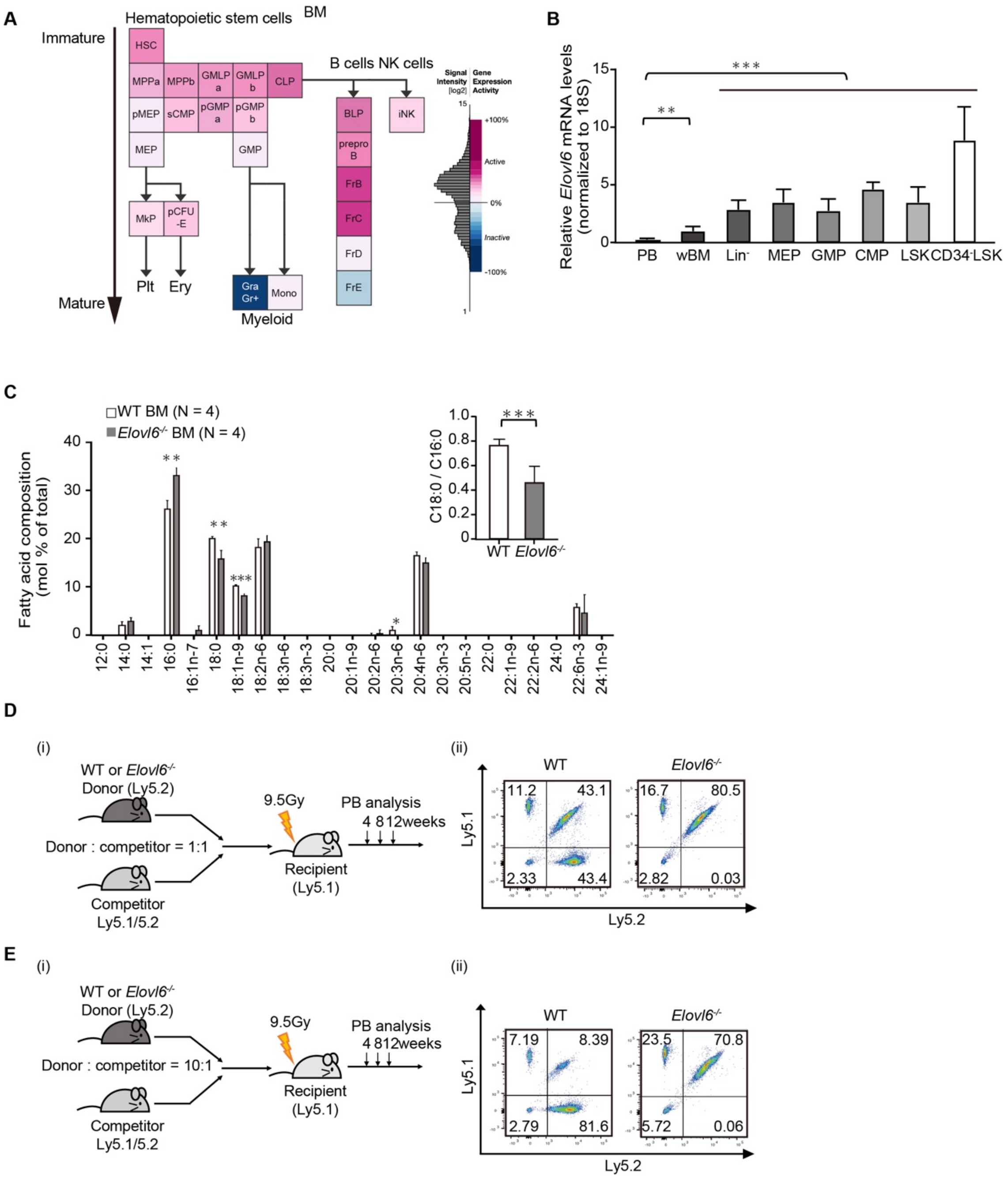

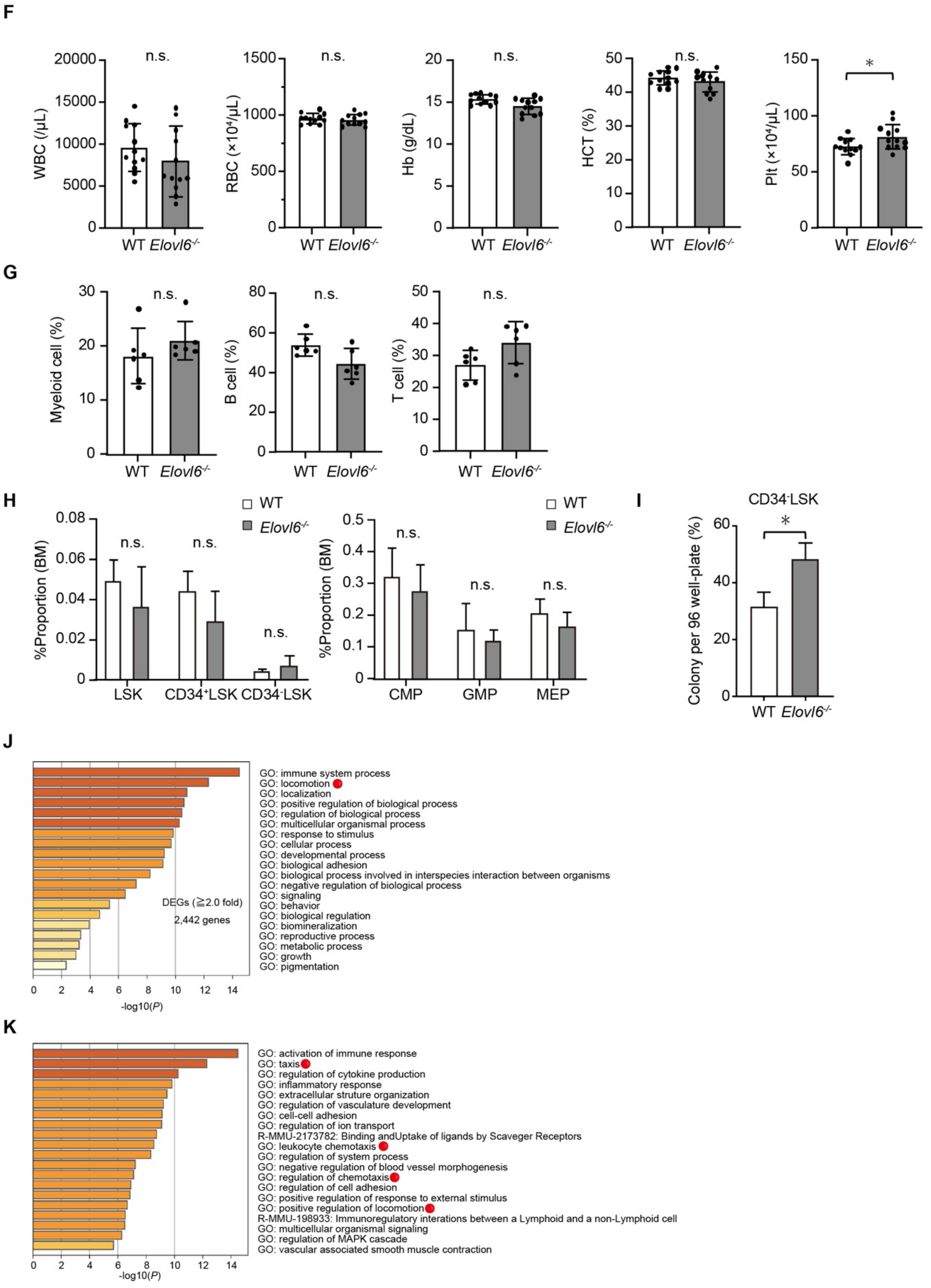

Figure S1.

A. *Elovl6* expression levels from 23 distinct mouse hematopoietic stem cell (HSC), progenitor, and differentiated cells in the bone marrow (BM) using the Gene Expression

Commons tool. Each box represents a defined cell population in the hematopoietic process (pink, increased expression; blue, decreased expression). Populations include HSC; multi-potent progenitor subset A (MPPa); MPPb; granulo/macrophage/lymphoid progenitor subset A (GMLPa); GMLPb; common lymphoid progenitor (CLP); premegakaryocyte/erythrocyte progenitor (pMEP); strict common myeloid progenitor (sCMP); pregranulocyte/macrophage progenitor subset A (pGMPa); pGMPb; megakaryocyte/erythrocyte (MEP); granulocyte/macrophage progenitor (GMP); megakaryocyte progenitor (MkP); preCFU-E (pCFU-E); platelets (Plt); erythrocyte (Ery); granulocyte (Gra); monocyte (Mono); earliest B-lymphoid progenitor (BLP); preproB cells (preproB); fraction B B-cell (FrB); fraction C B-cell (FrC); fraction D B-cell (FrD); fraction E B-cell (FrE); T1 B-cell (T1B); T2 B-cell (T2B); marginal zone B-cell (MzB); follicular B-cell (FoB); intermediate natural killer cell (iNK); mature natural killer cell (mNK); double negative T-cell (DN); double positive T-cell (DP); bone marrow (BM); and Spleen (Spl).

B. *Elovl6* mRNA levels in whole peripheral blood (PB), bone marrow (BM), hematopoietic progenitor cell (HPC), and HSC fractions; n = 4–5.

C. Fatty acid (FA) variants in BM cell lysates from wild-type (WT) and *E6KO* mice. The C18:0/C16:0 ratio is shown in the inset.

D. Outline of competitive repopulation assay (i). Representative flow cytometry data (ii) Experiments with 1×10^6 Ly5.2 WT (n = 9) or *E6KO* (n = 15) test cells and 1×10^6 Ly5.1/5.2 competitor BM cells are presented.

E. Outline of competitive repopulation assay (i). Representative flow cytometry data (ii). Experiments with 2×10^6 Ly5.2 WT (n = 9) or *E6KO* (n = 15) test cells and 2×10^5 Ly5.1/5.2 competitor BM cells are presented.

F. PB composition in wild-type (WT) or E6KO mice; n = 12, respectively.

G. Proportions of mature myeloid cells (Mac-1$^+$ or Gr-1$^+$ cells), T lymphocytes (CD3$^+$ cells), and B lymphocytes (B220$^+$ cells) in WT or E6KO mice at 8–16 weeks of age, as determined using flow cytometry; n = 6, respectively.

H. Frequency of Lin$^-$Sca1$^+$c-kit$^+$ (LSK), CMP, GMP, and MEP in BM of WT or E6KO mice; n = 4 each.

I. Colony formation by WT and *E6KO* CD34$^-$LSK cells; n = 3 each.

J. Differentially expressed genes (DEGs) in WT and *E6KO* CD34$^-$LSK cells. DEGs were screened for a fold-change ≥ 2.0. The enrichment analysis was performed using Metascape. Bar graph of enriched terms of DEGs (colored by *P*-values).

K. Significant enrichment terms of differentially expressed genes (≥ 2.0-fold) in WT versus *E6KO* $CD34^-$LSK cells, based on Metascape analysis of biological processes, colored by *P*-values.

Data are shown as the mean ± SD in all bar charts. **P* < 0.05, ***P* < 0.01, ****P* < 0.001.

Figure S2

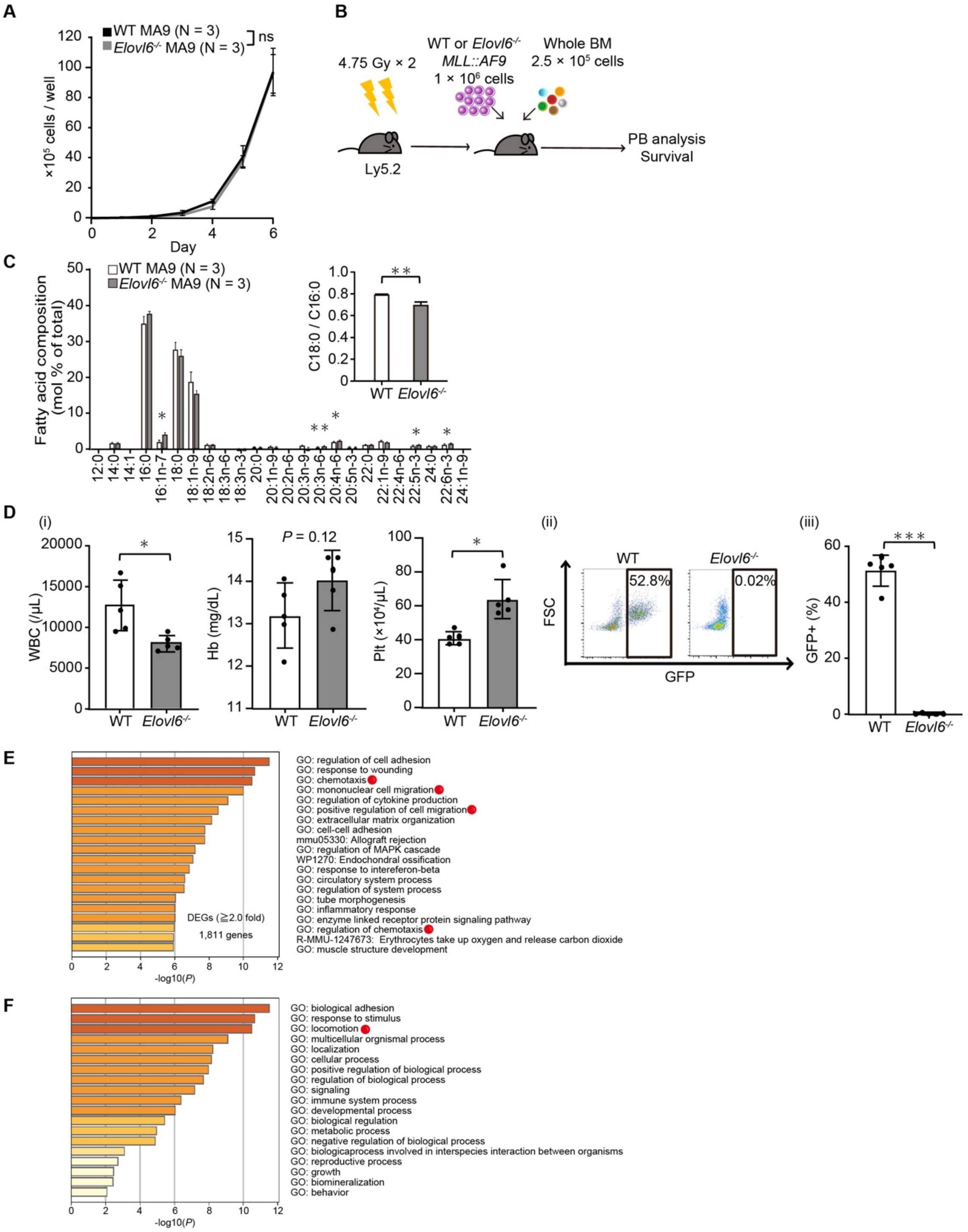

Figure S2.

A. Proliferation of MA9 cells in the presence of IL-3. Representative data from three independent experiments are shown.

B. Experimental design for a murine MA9-driven AML model.

C. Fatty acid (FA) variants in wild-type (WT) and *E6KO* MA9 cell lysates. Inset shows the

C16:0/C18:0 ratio.

D. White blood cell (WBC) counts, Hb levels, Plt counts (i), representative flow cytometry (ii), and chimerism (iii) of GFP^+ cells in PB of recipient mice 4 weeks after transplantation are presented; n = 5 each.

E. Differentially expressed genes (DEGs) in WT vs. *E6KO* MA9 cells. DEGs were screened for fold-change ≥ 2.0. The enrichment analysis was performed using Metascape. Bar graph of enriched terms of DEGs (colored by *P*-values).

F. Significant enrichment terms of DEGs (≥ 2.0-fold) in WT vs. *E6KO* MA9 cells, based on Metascape analysis of biological processes, colored by *P*-values.

Figure S3

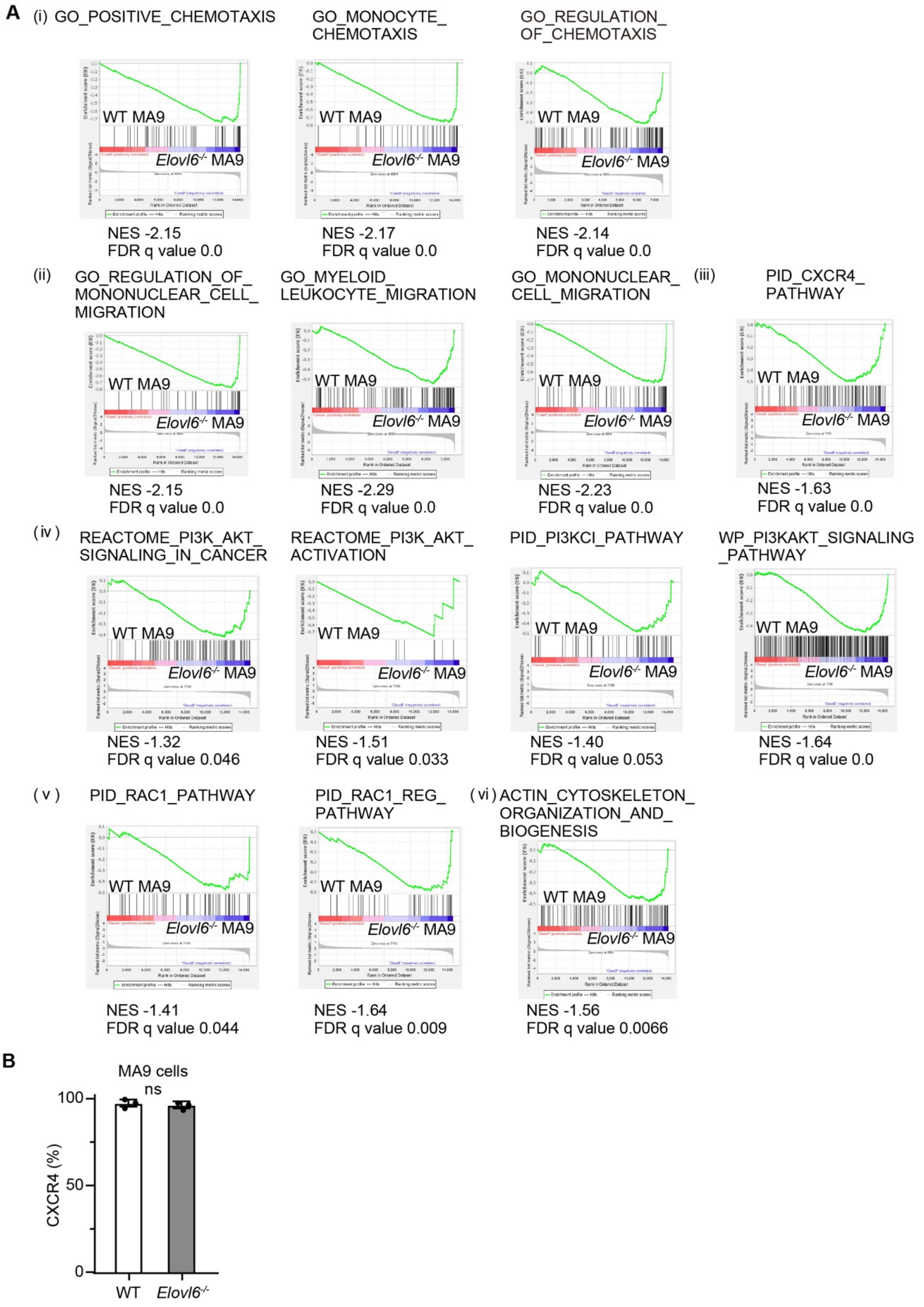
A
(i) GO_POSITIVE_CHEMOTAXIS
WT MA9
Elovl6-/- MA9
NES -2.15
FDR q value 0.0
GO_MONOCYTE_CHEMOTAXIS
WT MA9
Elovl6-/- MA9
NES -2.17
FDR q value 0.0
GO_REGULATION_OF_CHEMOTAXIS
WT MA9
Elovl6-/- MA9
NES -2.14
FDR q value 0.0
(ii) GO_REGULATION_OF_MONONUCLEAR_CELL_MIGRATION
WT MA9
Elovl6-/- MA9
NES -2.15
FDR q value 0.0
GO_MYELOID_LEUKOCYTE_MIGRATION
WT MA9
Elovl6-/- MA9
NES -2.29
FDR q value 0.0
GO_MONONUCLEAR_CELL_MIGRATION
WT MA9
Elovl6-/- MA9
NES -2.23
FDR q value 0.0
(iii) PID_CXCR4_PATHWAY
WT MA9
Elovl6-/- MA9
NES -1.63
FDR q value 0.0
(iv) REACTOME_PI3K_AKT_SIGNALING_IN_CANCER
WT MA9
Elovl6-/- MA9
NES -1.32
FDR q value 0.046
REACTOME_PI3K_AKT_ACTIVATION
WT MA9
Elovl6-/- MA9
NES -1.51
FDR q value 0.033
PID_PI3KCI_PATHWAY
WT MA9
Elovl6-/- MA9
NES -1.40
FDR q value 0.053
WP_PI3KAKT_SIGNALING_PATHWAY
WT MA9
Elovl6-/- MA9
NES -1.64
FDR q value 0.0
(v) PID_RAC1_PATHWAY
WT MA9
Elovl6-/- MA9
NES -1.41
FDR q value 0.044
PID_RAC1_REG_PATHWAY
WT MA9
Elovl6-/- MA9
NES -1.64
FDR q value 0.009
(vi) ACTIN_CYTOSKELETON_ORGANIZATION_AND_BIOGENESIS
WT MA9
Elovl6-/- MA9
NES -1.56
FDR q value 0.0066
B
MA9 cells
ns
100
50
0
CXCR4 (%)
WT
Elovl6-/-

C

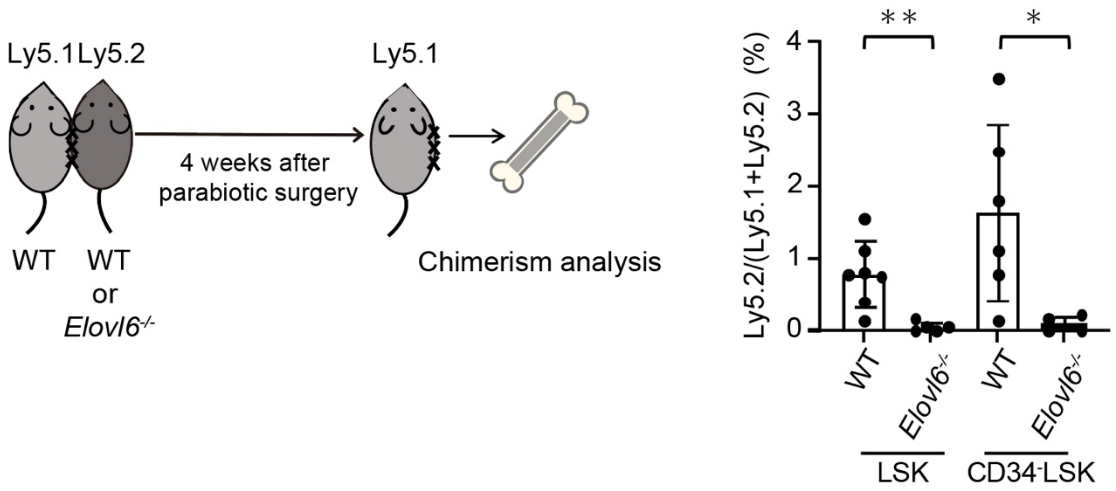

D

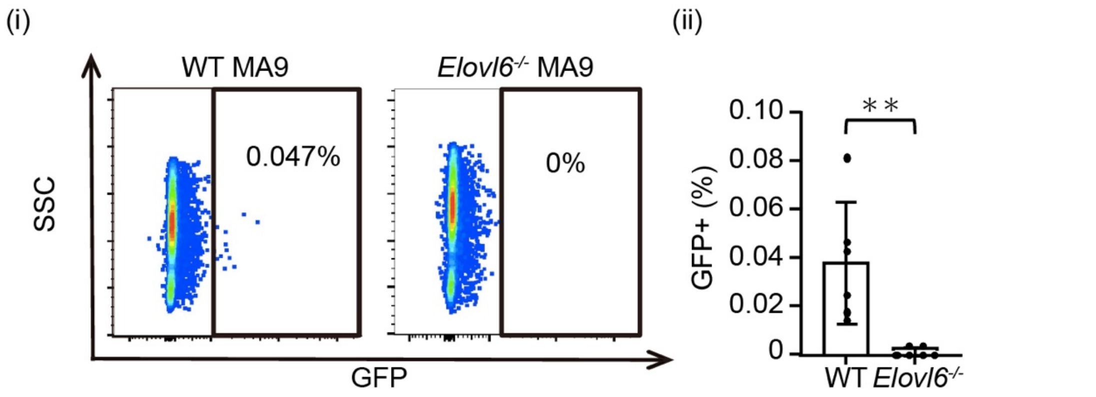

Figure S3.

A. Transcriptome analysis of wild-type (WT) and *E6KO* MA9 cells. Gene set enrichment analysis (GSEA) for pathways related to (i) chemotaxis, (ii) migration, (iii) CXCR4 pathway, (iv) PI3K-AKT signaling, (v) RAC signaling, and (vi) actin-cytoskeleton. Normalized enrichment scores (NES) and FDR *q*-values are indicated.

B. Percentage of CXCR4-positive cells among WT or *E6KO* MA9 cells, as analyzed using flow cytometry with an anti-CXCR4 antibody. Data are shown as the mean ± SD in all bar charts.

C. Outline and results of parabiotic study. Chimerism of BM Ly5.2 LSK and $CD34^-$LSK cells in each Ly5.1 parabiont; WT (n = 6–7) or *E6KO* (n = 4–5).

D. Homing assay. (i) Representative flow cytometry results. (ii) Chimerism of WT or *E6KO* GFP^+ cells in BM mononuclear cells. Summary of six independent experiments; n = 6 each.

Data are shown as the mean ± SD in all bar charts. *$P < 0.05$, **$P < 0.01$, ***$P < 0.001$.

Figure S4

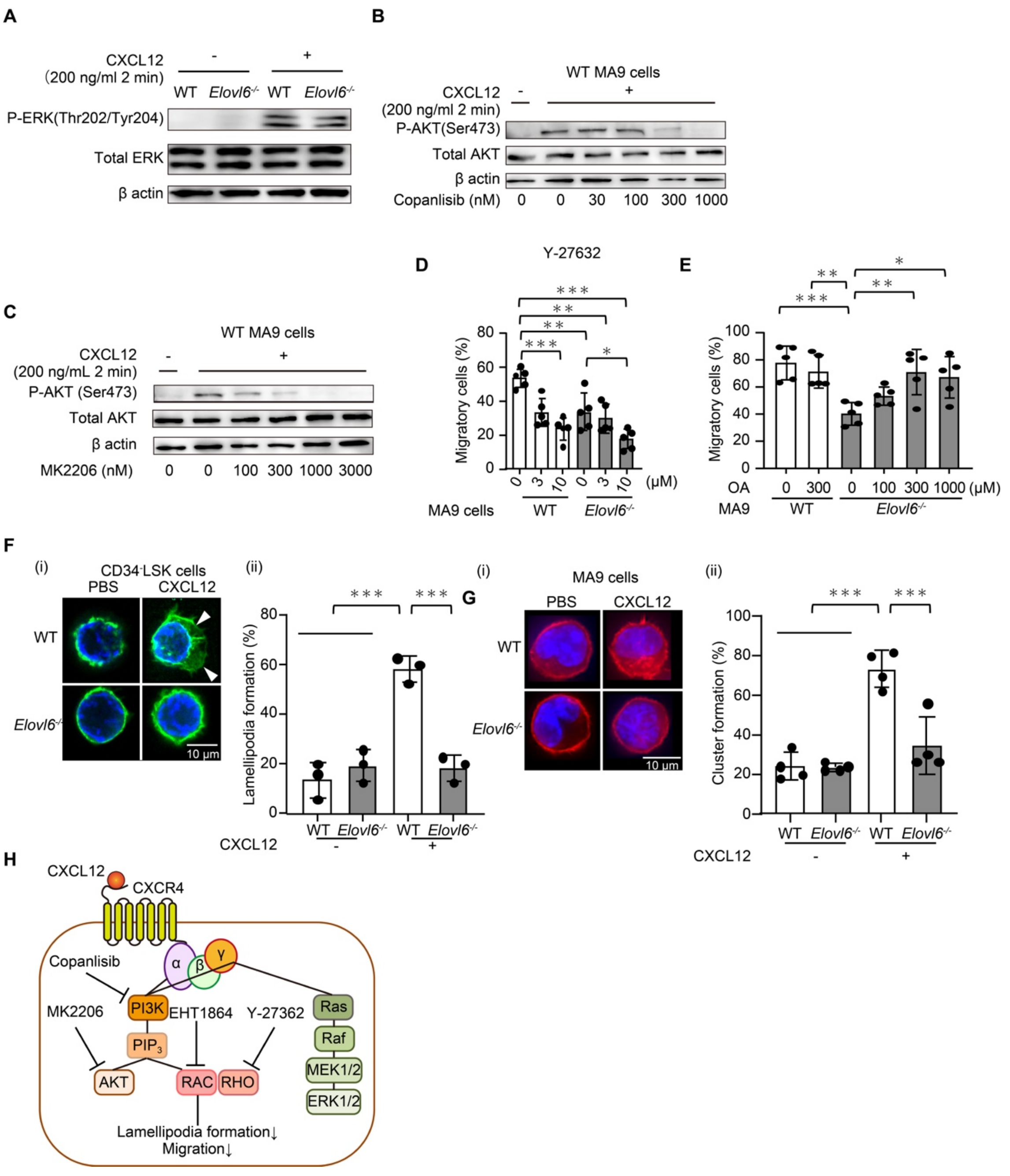

Figure S4.

A. Phosphorylation of ERK in WT or *E6KO* MA9 cells before and after CXCL12 stimulation. Representative western blot.

B. Western blot analysis showing the effects of Copanlisib on AKT phosphorylation in WT MA9 cells.

C. Western blot analysis showing the effects of MK2206 on AKT phosphorylation in WT MA9 cells.

D. Effect of Y-27632 on migration of WT or *E6KO* MA9 cells toward a CXCL12-containing chamber; n = 5 each.

E. Effect of oleic acid (OA) addition (0–1000 μM) on migration of WT or *E6KO* MA9 cells toward a CXCL12-containing chamber; n = 5 each.

F. Actin remodeling in WT or *E6KO* $CD34^-$LSK cells ((i) and (ii)) after CXCL12 stimulation. Representative immunostaining for F-actin (green) and DAPI (nuclei; blue) (i) and frequency of lamellipodia formation (ii) in $CD34^-$LSK cells (n = 30 cells) are shown. Arrowheads indicate lamellipodia formation in response to CXCL12 stimuli.

G. F-actin staining of representative single WT MA9 or *E6KO* MA9 cells stimulated for 30 min with CXCL12, suggesting that CXCL12 induces F-actin cluster formation. F-actin clusters were stained with phalloidin (red), and nuclei were stained with DAPI (blue). (ii) Statistical analysis of frequency of cluster formation in MA9 cells (n = 50 cells) shown in the left panel.

H. Schema summarizing the CXCL12-CXCR4-PI3K-AKT/RAC pathway and targets of inhibitors.

Data are shown as the mean ± SD in all bar charts. *$P < 0.05$, **$P < 0.01$, ***$P < 0.001$.

Figure S5

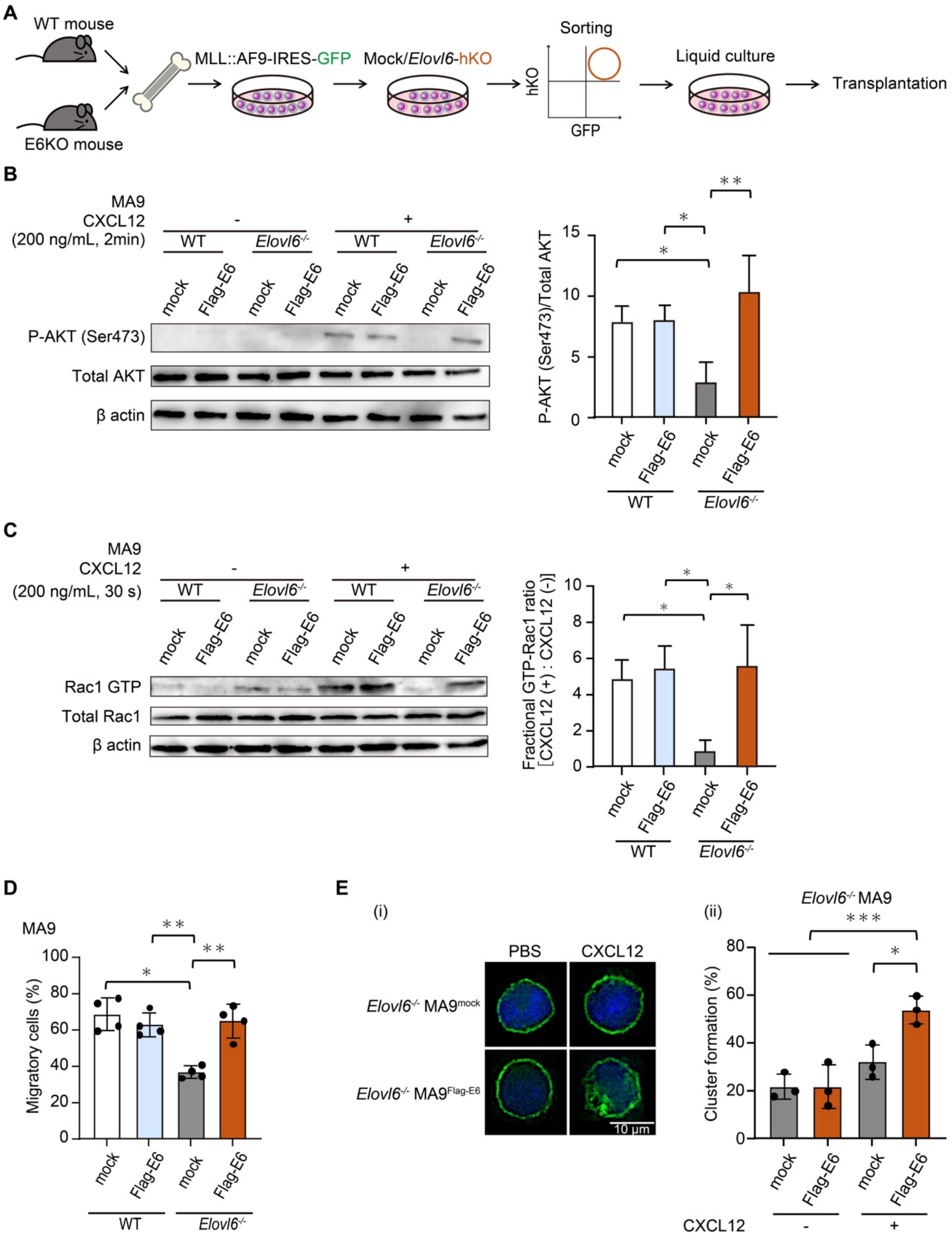

Figure S5.

A. Outline of the generation of *Elovl6* re-expressed/over-expressed MA9 cells.

B. (left) WT or *E6KO* MA9 cells infected with mock or Elovl6 hKO were treated for 2 min with CXCL12 and subjected to western blotting with the indicated antibodies.

(right) Analysis of the data shown on the left.

C. Pull-down assay for GTP-bound Rac1 in *E6KO* $MA9^{Flag\text{-}E6}$, *E6KO* $MA9^{mock}$, WT $MA9^{Flag\text{-}E6}$, and WT $MA9^{mock}$ cells before and after CXCL12 stimulation. Representative western blot (i) and results of four experiments (ii).

D. Transwell chemotaxis assay of *E6KO* $MA9^{Flag\text{-}E6}$, *E6KO* $MA9^{mock}$, WT $MA9^{Flag\text{-}E6}$, and WT $MA9^{mock}$ cells toward a CXCL12-containing chamber; n = 4 each.

E. F-actin staining of representative single *E6KO* MA9 cells infected with mock or Elovl6 hKO and stimulated for 30 min with CXCL12. F-actin clusters were stained with phalloidin green and nuclei were stained with DAPI (blue).

(ii) Statistical analysis of frequency of cluster formation in MA9 cells (n = 50 cells).

Data are shown as the mean ± SD in all bar charts. *P < 0.05, **P <0.01, ***P < 0.001.

Figure S6

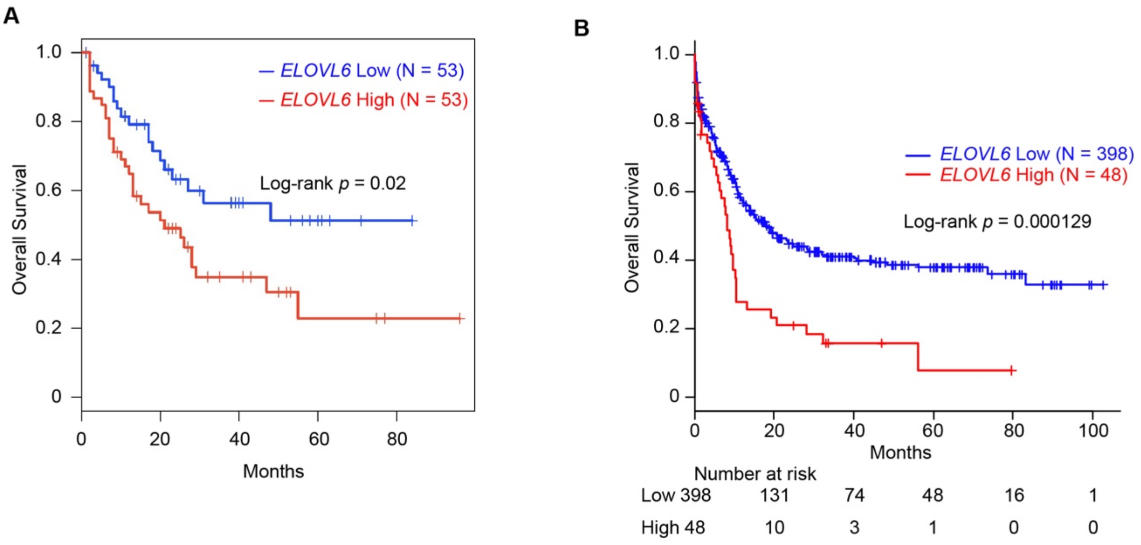

Figure S6.

A. Kaplan-Meier curves of the overall survival of 106 AML patients in The Cancer Genome Atlas data set (http://gepia2.cancer-pku.cn) classified by mean *ELOVL6* mRNA levels of their BM cells.

B. Kaplan–Meier curves of the overall survival of 446 AML patients in the Beat AML dataset (http://www.vizome.org).

Statistical analysis was performed using the log-rank test.

Figure S7

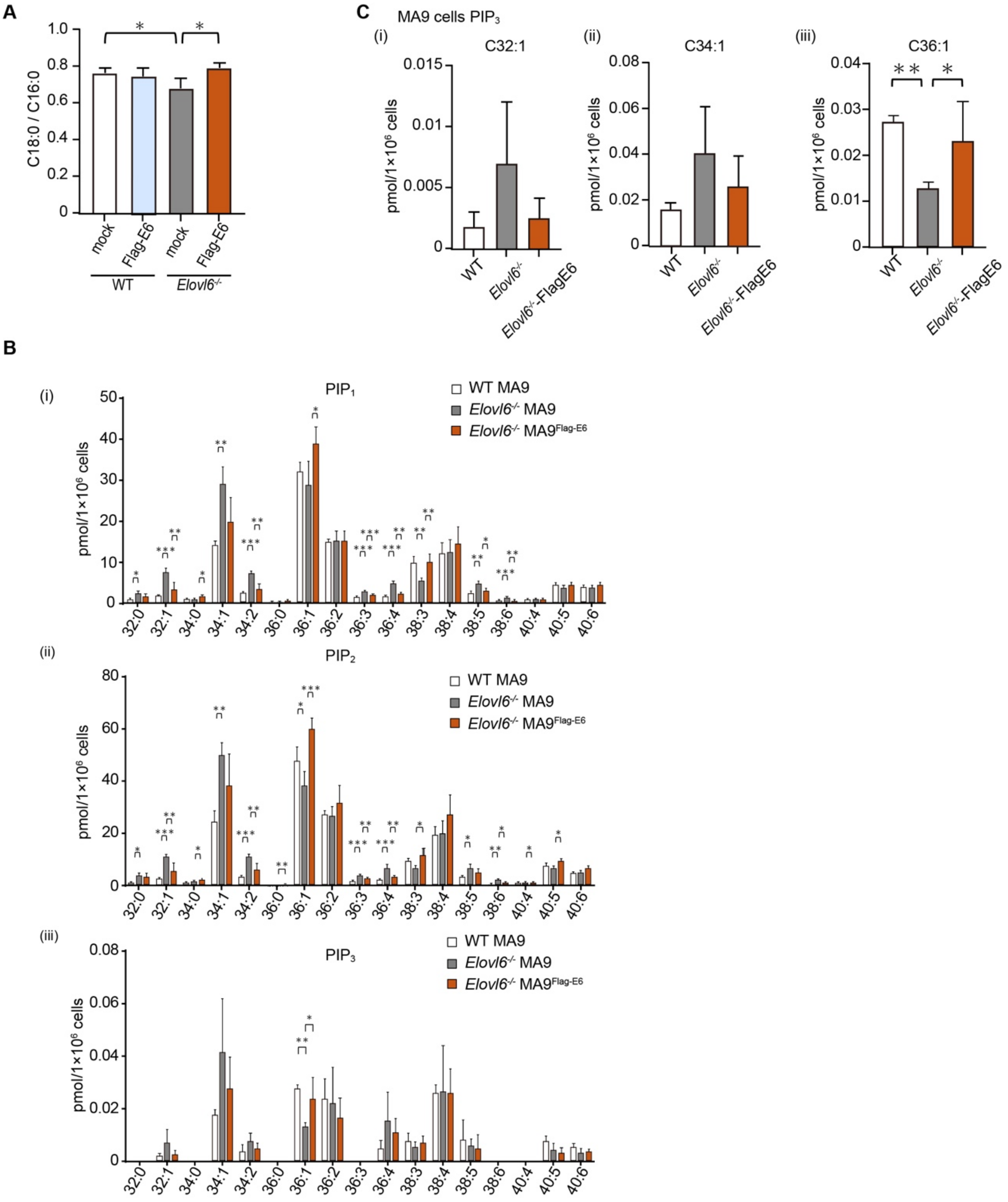

Figure S7.

A. The C18:0/C16:0 ratio in *E6KO* MA9$^{Flag-E6}$, *E6KO* MA9^{mock}, wild-type (WT) MA9$^{Flag-E6}$, and WT MA9^{mock} cell lysates; n = 4 each.

B. Liquid chromatographic–tandem mass spectrometric analysis of MA9 cell PIPs from wild-type (WT) MA9, *E6KO* MA9, or *E6KO* MA9$^{Flag-E6}$ cells (n = 4 per group). Shown are PIP_1 (i), PIP_2 (ii), and PIP_3 levels (iii).

C. Liquid chromatographic–tandem mass spectrometric analysis of MA9 cell PIP_3 from WT MA9, *E6KO* MA9, or *E6KO* $MA9^{Flag\text{-}E6}$ cells (n = 4 per group). Shown are C32:1 (i), C34:1 (ii), and C36:1 (iii).

Figure S8

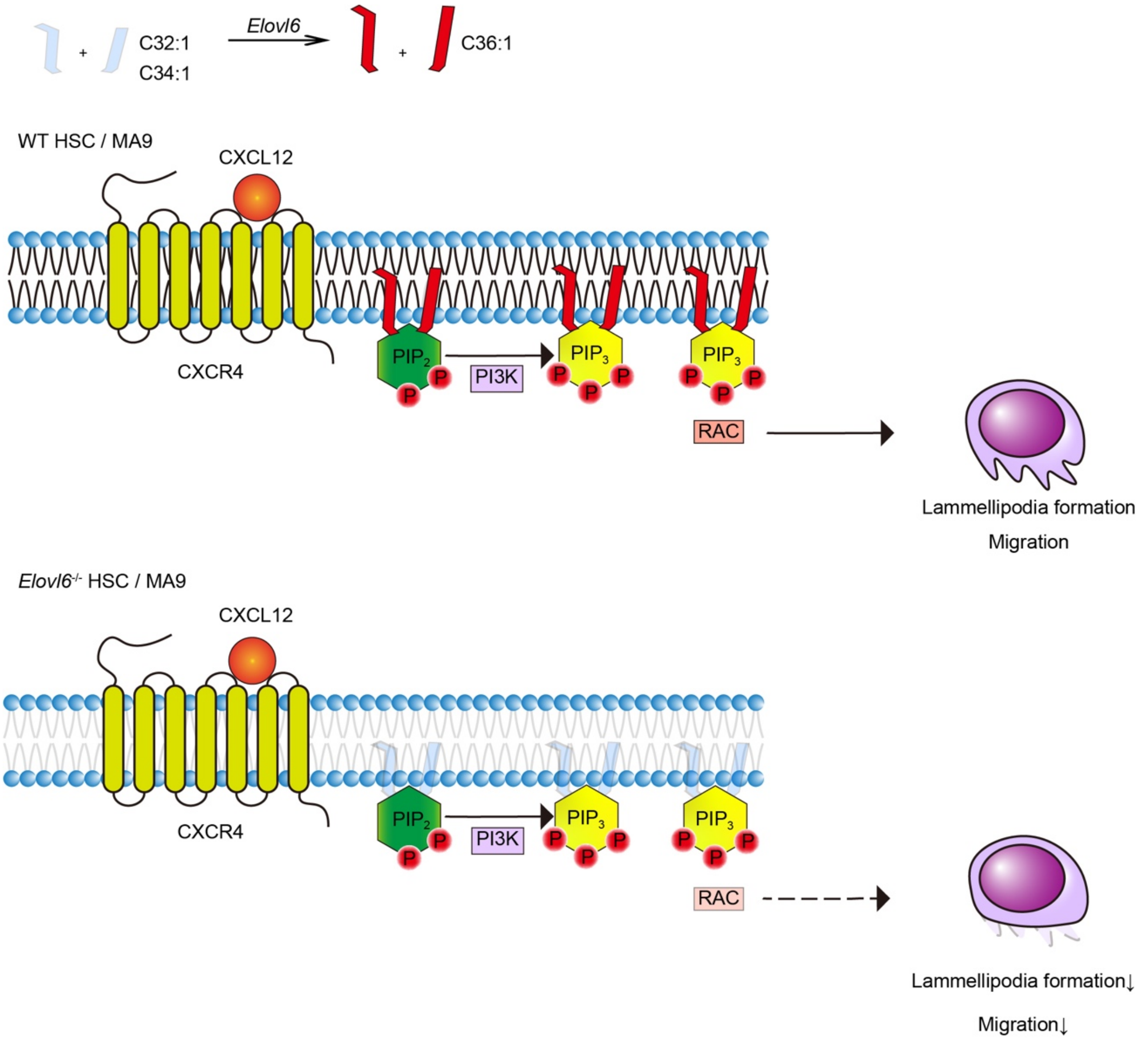

Figure S8. Proposed model depicting the role of the *Elovl6*-mediated PI3K-RAC pathway.

Supplementary Table 1. Primer sequences used in this study

Related to		Sequence
Genotype PRIMERS	Elovl6 LEXKO Fw	5′-AGGCCAGAGGTATTGAATCACC-3′
	Elovl6 LEXKO Rv	5′-GACATCATTACTCACTCCAGCC-3′
	Elovl6 LTR Rv	5′-ATAAACCCTCTTGCAGTTGCATC-3′
RT-qPCR PRIMERS	Elovl6 Fw	5′-CCCGAACTAGGTGACACGAT-3′
	Elovl6 Rv	5′-CCAGCGACCATGTCTTTGTA-3′
